# Adipose-Derived Mesenchymal Stem Cells Combined With Extracellular Vesicles May Improve Amyotrophic Lateral Sclerosis

**DOI:** 10.3389/fnagi.2022.830346

**Published:** 2022-05-18

**Authors:** Xichen Wang, Yong Zhang, Tian Jin, Benson O. A. Botchway, Ruihua Fan, Lvxia Wang, Xuehong Liu

**Affiliations:** ^1^Department of Histology and Embryology, School of Medicine, Shaoxing University, Shaoxing, China; ^2^Institute of Neuroscience, Zhejiang University School of Medicine, Hangzhou, China; ^3^School of Life Sciences, Shaoxing University, Shaoxing, China

**Keywords:** adipose-derived mesenchymal stem cells, amyotrophic lateral sclerosis, neuronal injury, combinational therapy, extracellular vesicles

## Abstract

The complexity of central nervous system diseases together with their intricate pathogenesis complicate the establishment of effective treatment strategies. Presently, the superiority of adipose-derived mesenchymal stem cells (ADSCs) on neuronal injuries has attracted significant attention. Similarly, extracellular vesicles (EVs) are potential interventional agents that could identify and treat nerve injuries. Herein, we reviewed the potential effects of ADSCs and EVs on amyotrophic lateral sclerosis (ALS) injured nerves, and expound on their practical application in the clinic setting. This article predominantly focused on the therapeutic role of ADSCs concerning the pathogenesis of ALS, the protective and reparative effects of EVs on nerve injury, as well as the impact following the combined usage of ADSCs and EVs in ALS.

## Introduction

Amyotrophic lateral sclerosis (ALS) is a neurodegenerative disease. It is characterized by dysfunction of the upper and lower motor neurons (Hardiman, 2021). Clinical manifestations of ALS include adult-onset focal muscle weakness and emaciation, usually beginning in the muscles of the extremities and spreading as the disease progression. Some patients have different degrees of non-motor performance, including cognitive and behavioral changes (Phukan et al., 2007; Masrori and Van Damme, 2020). ALS is difficult to diagnose due to the high variability of clinical manifestations. Presently, its diagnosis is based on clinical signs and electromyography. In addition, ultrasound and magnetic resonance imaging are auxiliary methods that assist with disease diagnosis (Goedee et al., 2020; Johnsen, 2020). ALS patients have poor prognosis, and most ALS patients eventually die due to respiratory dysfunction. Although no medical intervention for the complete alleviation of ALS presently exists, studies have shown that non-invasive ventilation and medication (riluzole and edaravone) improve patients’ quality of life and delay disease progression (Niedermeyer et al., 2019; Chiò et al., 2020). As a heterogeneous syndrome, the pathogenesis of ALS is still unclear. However, genetic abnormalities often cause changes in their corresponding molecular mechanisms (Blokhuis et al., 2013). ALS pathogenesis includes cell function aberrancy and changes regarding the molecular content (van Es et al., 2017). SOD1^*G*93*A*^, as a marker protein of the ALS, activates NLRP3 inflammasome in microglia via its promotion of IL-1β secretion, which in turn leads to neuroinflammation and neurotoxicity (Deora et al., 2020). Studies have shown that ERK1/2 kinase can drive accumulated TAR DNA binding protein 43 (TDP-43) to the cytoplasm. Aggregated TDP-43 binds to specific microRNAs, resulting in interruption of protein synthesis. This causes the overall imbalance of mitochondria and aggravates oxidative stress. The decrease of glutathione content in cytoplasm is significant to the occurrence of oxidative stress response (Romano et al., 2020; Zuo et al., 2021). Furthermore, glutamate transporter deficiency increases the content of extracellular glutamate, which culminates in the degeneration of motor neurons (Bonifacino et al., 2019).

The diversity of ALS pathogenesis has provided new ideas for the treatment of the disease. In recent years, mesenchymal stem cells (MSCs) and extracellular vesicles (EVs) from different sources have played a significant role in neurodegenerative diseases. MSCs, as pluripotent and self-renewing stem cells, have a wide range of sources, can be obtained from bone marrow and adipose tissue, and be easily expanded *in vitro* (Gugliandolo et al., 2019). Transplanted MSCs mitigated the autophagy and apoptosis of motor neurons, produced neuroprotective effects and prolonged life span in the models of the ALS mice. More interestingly, the repeated injection did enhance these effects (Řehořová et al., 2019). Several routes can achieve MSCs in ALS treatment: One is the obstruction of the disease by replacing and protecting the damaged neurons (Gao et al., 2019). Through its paracrine effects, MSCs can release nutritional factors to maintain the microenvironment of neurons (Sykova et al., 2021). Obviously, the extraction of MSCs is more convenient from adipose tissue (Strioga et al., 2012). In addition, its acquisition does not involve ethical issues like that of embryonic mesenchymal stem cells (Gugliandolo et al., 2019). Therefore, adipose-derived mesenchymal stem cell transplantation is a potential cell therapy for ALS (Marote et al., 2016).

Extracellular vesicles is a tiny vesicle that can be secreted by various cells in the body, and is formed after induction of plasma membrane and formation of intracellular multivesicular bodies. These are secreted to the outside of the cell by exocytosis, and transfer nucleic acids, proteins and lipids between cells, acting on target cells to induce their function and effect (He et al., 2018; Kalluri and LeBleu, 2020). EVs come from a wide range of sources in the human body, and mediate communications and maintenance of cells (Doyle and Wang, 2019). Studies have demonstrated that endothelial cells, glial cells, and MSCs can produce EVs (Frühbeis et al., 2013; Song H. et al., 2019; Gonçalves et al., 2020), and exists in plasma, urine, saliva and cerebrospinal fluid (Elsharkawi et al., 2019; Jain et al., 2019; Rahman et al., 2019; Mi et al., 2020). More importantly, they can protect and repair nerve injury. This article reviews the potential effects of ADSCs and ADSCs EVs in the treatment of ALS. In addition, the therapeutic effects focusing on ADSCs EVs and ADSCs delivery pathways and the benefits associated with repeated delivery will be described.

## The Advantages of Adipose-Derived Mesenchymal Stem Cells Transplantation

Adipose tissue is abundant in the human body, thus, obtaining ADSCs is convenient for autologous transplantation (Marconi et al., 2013). The safety of autologous ADSCs for the treatment of ALS has been reported in a clinical study (Staff et al., 2016). ADSCs protect and repair nerves through differentiation and paracrine. In comparison to bone marrow mesenchymal stem cells (BMSCs), ADSCs can secrete more glial cell-derived neurotrophic factor (GDNF) and fibroblast growth factor 2 (Otsuka et al., 2021). Noteworthy is that GDNF can promote axonal growth and the formation of functional synapses, while fibroblast growth factor 2 can enhance the survival, proliferation and differentiation of nerve cells (Zhu S. et al., 2020; Shamadykova et al., 2021). Regarding the extraction of ADSCs from different parts of the body, several differences exist. For instance, subcutaneous ADSCs express higher levels of fibronectin and vimentin, which contributes to their directional migration. The expression of stemness-associated genes, such as c-MYC, SOX2, KLF4, and NANOG in visceral ADSCs are significantly upregulated, indicating that visceral ADSCs have better differentiation ability than subcutaneous ADSCs (Ritter et al., 2019). The subsequent sections will explicate some of the biological functions of ADSCs transplantation.

### Provision of Nutrition and Support

Previous studies have shown that ADSCs can protect neurons in neurological diseases, such as traumatic brain injury and cerebral ischemia, reduce secondary injury and improve neurological function (Ma et al., 2019; Paudyal et al., 2021). ADSCs can improve nutrition and support the survival of neurons in two ways. (1) ADSCs can be differentiated into neural cells to enhance the effects of nerve cell loss in the injured area. Notably, ADSCs obtained from dogs showed immense ability in differentiating into neurogenic lineage (Roszek et al., 2017). Also, a study showed that ADSCs can undergo neural differentiation induced by olfactory ensheathing cells or Schwann cell cultures (Lo Furno et al., 2018). ADSCs differentiated into neural lineage can highly express markers of neurons and glial cells, such as neurofilament H and glial fibrillary acid protein (Prpar Mihevc et al., 2020). The neural differentiation potential of ADSCs under different inducers also indicates that ADSCs could differentiate into different types of nerve cells in complex living environment *in vivo*. ADSCs transplantation can hinder myelin degeneration to promote increased number of myelin fibers in the injured area of the peripheral nerve, and enhance the regeneration of neurons in spinal ganglion (Masgutov et al., 2019). Moreover, ADSCs can differentiate into Schwann-like cells and improve the regeneration of motor neurons in the repair of peripheral nerve injury. In addition, undifferentiated ADSCs can secrete growth factors and have a synergistic effect with Schwann cells (Kingham et al., 2014). (2) Transplanted ADSCs secrete nerve growth factors, BDNF, and GDNF. BDNF-tyrosine receptor kinase B (TrkB) is an important mechanism that maintains neuromuscular junction (NMJ) stability. Thus, decreased activation of TrkB may reduce the signal transduction in cells, resulting in the instability of NMJ, and subsequently leading to motor neuronal death (Just-Borràs et al., 2019). Besides, transplanted ADSCs can secrete BDNF (Kim et al., 2014). BDNF can augment the signal transduction of BDNF-TrkB in neuronal cells to maintain normal NMJ and delay ALS progression (Just-Borràs et al., 2019). Interestingly, BDNF-TrkB signal transduction promoted the proliferation of oligodendrocyte precursor cells, culminating in its increment and repair of myelin sheath (Geraghty et al., 2019). And the combination of ADSCs with GDNF enhances the survival of ADSCs and increases their ability to differentiate into neural-like cells. This implies that the sustained release of GDNF may be the reason for the pluripotent differentiation of ADSCs (Sun S. et al., 2020). CRISPR activation-engineered ADSCs can activate endogenous neurotrophic factor genes to induce the expressions of BDNF, GDNF, and nerve growth factors. These neurotrophic factors, in turn, can stimulate the proliferation of neurons and enhance the growth of neurites (Hsu et al., 2019). Furthermore, ADSCs can directly secrete neurotrophic factors or indirectly regulate local glial cells, and transform them into neuroprotective secretory phenotypes (Marconi et al., 2013). Following ADSCs transplantation, BDNF and GDNF expressions in the neural microenvironment are upregulated. BDNF and GDNF do significantly improve the survival environment of neurons. In addition, the stimulation of ADSCs with a combination of growth factors promoted the secretion of vascular endothelial growth factor-A and angiopoietin-1. These secretions led to enhanced axonal and blood vessel regeneration. More importantly, in the 4th week after the transplantation of ADSCs, TUNEL positive cells are significantly decreased, indicating that various factors produced by ADSCs could block apoptosis of motor neurons (Kim et al., 2014). Following the injection of green fluorescent protein labeled ADSCs, inflammation within the spinal cord was observed (Constantin et al., 2009; Marconi et al., 2013). This suggests that ADSCs could locate the site of injury. Therefore, ADSCs may improve the microenvironment of neuronal survival by directly or indirectly secreting regulatory factors, and stimulating their own neural differentiation. The differentiation and secretion of ADSCs have significant nutritional and supportive effects on nerve cells.

### Inhibition of Inflammatory Response

Adipose-derived mesenchymal stem cells can secrete a variety of anti-inflammatory factors, inhibit the activation of inflammatory cells, and repress the secretion of pro-inflammatory factors. Also, ADSCs promote the transformation of microglia and other cells to neuroprotective phenotype that in turn protects motor neurons from the influence of inflammatory environment and prevents motor neuronal degeneration and death.

Inflammatory markers, interleukin-1β (IL-1β), IL-6, IL-8, tumor necrosis factor-α (TNF-α), and TNF receptor-1, are significantly increased in ALS patients (Hu et al., 2017). Furthermore, the number of activated astrocytes and activated microglia increases in the damaged area of ALS. Also, there are inflammatory cells within the periphery, such as lymphocytes, mast cells, dendritic cells, and neutrophils, but the correlation between these inflammatory cells is not clear (Trias et al., 2018; McCauley and Baloh, 2019; McCauley et al., 2020). Astrocytes and microglia are key players in neuroinflammation. In particular, microglia release dysfunctional or broken mitochondria into the environment, which causes activated astrocytes to spread inflammation. The spread of inflammation leads to the degeneration and death of neurons (Joshi et al., 2019). Therefore, the inhibition and alleviation of the activation of glial cells and their signaling pathway can effectively reduce the occurrence of neuroinflammation.

Adipose-derived mesenchymal stem cells treatment downregulates the expression of inflammatory cytokines and the number of Iba1^+^ microglia, implying that ADSCs suppress microglia activation (Huang X. et al., 2020). Moreover, ADSCs induce microglia phenotype transformation from M1 to M2, and minimizes the release of pro-inflammatory factors (Angeloni et al., 2020). M2 microglia can secrete interleukin (IL)-4, IL-10, and IL-13. These pro-inflammatory factors play an anti-inflammatory role and can alleviate immune responses (Lyon et al., 2019). Moreover, the expressions of BDNF and TrkB are downregulated due to inflammation. However, ADSCs transplantation reverses these effects (Huang X. et al., 2020). BDNF-TrkB signal has been related to neuronal cell injury. The inhibition of this signal transduction may decrease the activated number of astrocytes and microglia to alleviate neuroinflammation (Ding et al., 2020). Monocytes/macrophages and CD3-positive lymphocytes at the site of neuroinflammation in ADSCs treated mice are markedly decreased (Marconi et al., 2012), implying that ADSCs can suppress neuroinflammation. The silencing of the nuclear factor erythroid 2-related factor 2 (Nrf2) expression downregulates HO-1 downstream expression, and increases the level of NLRP3 (Chen Z. et al., 2019). NLRP3 enhances IL-1β secretion, which leads to neuroinflammation (Deora et al., 2020). Also, the expression of M2 microglia markers, Ym1 and Arg1, were increased after treatment with overexpressed-Nrf2 ADSCs (Huang X. et al., 2020). The above studies demonstrate that ADSCs could reverse nerve cell injuries and release inflammatory factors via the Nrf2/HO-1 signaling pathway.

### Curtailment of Oxidative Stress Response

**FIGURE 1:**
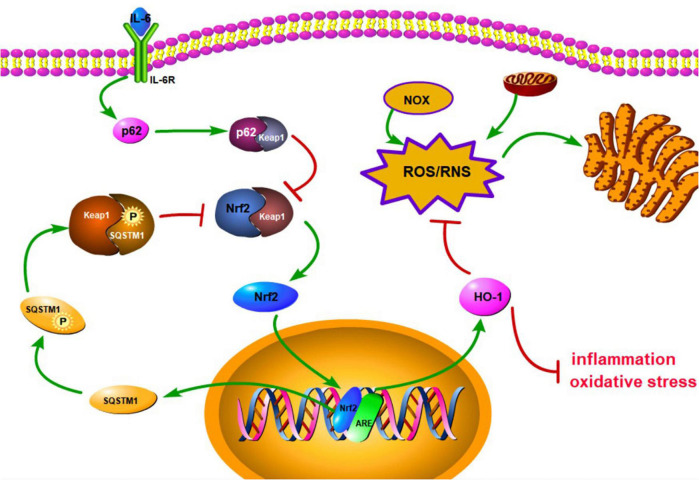
Schematic diagram of the activation of antioxidant stress signal pathway, Keap1/Nrf2/HO-1. IL-6 can bind to membrane surface receptors and activate p62 to bind to Keap1, thereby inhibiting the binding of Keap1 to Nrf2. Subsequently, keap1-Nrf2 complex releases Nrf2 to enter the nucleus and promote the expression of HO-1 and SQSTM1. HO-1 can inhibit inflammation and oxidative stress, and reduce endoplasmic reticulum stress by inhibiting ROS/RNS pathway. SQSTM1 can inhibit keap1-Nrf2 complex to result in releasing Nrf2 and form a positive feedback.

Oxidative stress is an important mechanism in ALS. ROS or reactive nitrogen species accumulated in cells can induce oxidative stress. ROS mainly comes from damaged mitochondria in the motor neurons of ALS patients (Wang et al., 2019). During ALS, the microglia in the central nervous system highly express NOX2, which is a source of ROS (Seredenina et al., 2016). In oxidative stress, increased reactive oxygen species (ROS) triggers the loss of motor neurons as well as the proliferation of glial cells. In addition, the increase of intracellular ROS content induces the aggregation of TDP-43 and damages the mitochondria (Ohta et al., 2019; Zuo et al., 2021). Because oxidative stress considerably impairs nerve cells in ALS, it is important to eliminate ROS/RNS. Interestingly, the activation of the Nrf2 triggers the expression of antioxidant enzymes (Munguía-Martínez et al., 2019). In the cytoplasm, the Nrf2 binds to Kelch-like ECH-associated protein 1 (Keap1) to form a complex that inhibits the Nrf2 activity. When the keap1-Nrf2 complex is stimulated by external IL-4 and IL-13, the binding of the Keap1 and the Nrf2, causing the release of the Nrf2 (Furue, 2020). The Nrf2 released into the cytoplasm gradually translocate to the nucleus. In the nucleus, the Nrf2 binds to the antioxidant response element (ARE) to activate downstream pathways (Zhang et al., 2019, 2020). In addition, phosphorylated SQSTM1 interacts with Keap1 to promote the release of Nrf2, which in turn upregulates the expression of Nrf2 targeted genes (Deng et al., 2020). ADSCs can express IL-6, and IL-6 combined with IL-6 receptor augments p62 expression in cells (Noman et al., 2020; Zhu et al., 2021). The increased p62 binds to Keap1, causing the release of Nrf2 (Sun Y. et al., 2020). The downregulation of Keap1 gene expression using the CRISPR/Cas9 system caused the release of Nrf2 from the outcome of ubiquitination and protein degradation. This provides a premise for Nrf2 to enter the nucleus. Immunofluorescence analysis did show the location of the Nrf2 to be the nucleus (Hu et al., 2020). Besides, increased nuclear Nrf2 can enhance the expression of antioxidant enzymes in ADSCs (Garrido-Pascual et al., 2020). MDA (an oxidative stress marker) level was decreased after Nrf2 expression in ADSCs (Hu et al., 2020), indicating the antioxidant capacity of Nrf2 and its downstream pathway. Interestingly, HO-1 expression was downregulated after the knockdown of Nrf2 expression, implying that HO-1 is a downstream product of Keap1/Nrf2. Moreover, the expression of endoplasmic reticulum stress-related protein is considerably increased after HO-1 knockout. This demonstrates that Nrf2/HO-1 pathway can inhibit endoplasmic reticulum stress and improve cell damage, while also curtailing ROS that is generated during oxidative stress (Xu B. et al., 2020). The schematic diagram of intracellular ROS elevation stimulating Keap1/Nrf2/HO-1 signaling pathway activation against oxidative stress is depicted in Figure 1. In the oxidative stimulation environment, ADSCs pretreated with 0.25 mM H_2_O_2_ expresses higher Nrf2 and related antioxidant enzymes than untreated ADSCs, resulting in better antioxidant effect. This shows that the complete expression of Nrf2 in ADSCs improves ADSCs own oxidative tolerance, while also promoting survival and proliferation. Moreover, it can counteract the secretion of oxidative stress factors (Garrido-Pascual et al., 2020). ADSCs can resist oxidative stress through Nrf2 and its downstream antioxidant proteins.

## The Protective and Reparative Effects of Extracellular Vesicles on Neuronal Injury

Extracellular vesicles from different sources have disparate degrees of protection against nerve injury. For instance, astrocyte-derived EVs delivered GJA1-20k to reduce the damage to mitochondrial structure caused by neuronal injury and mitigate the degree of neuronal apoptosis (Chen W. et al., 2020). EVs of oligodendrocytes can be internalized by neurons, which may enhance neuronal metabolism and survival under the regulation of cell stress. In addition, EVs derived from M2 microglia and Schwann cells can also maintain intracellular homeostasis and promote neuronal survival by transmitting miRNA (Song Y. et al., 2019; Gonçalves et al., 2020). Several studies have shown that ADSCs-derived EVs have more significant neuroprotective effects, and immunostaining analysis demonstrated that neurons preferentially ingest MSCs-derived EVs (Guo et al., 2019). Therefore, ADSCs-derived EVs may be used as a potential therapeutic mechanism for nerve injury. Also, EVs can be used as biomarkers to detect and treat diseases (Jain et al., 2019), especially in ALS (Table 1). IL-6 level in astrocytic-derived EVs in the central nervous system of ALS patients gradually increases with the development of the disease, indicating its potentiality in being a biological marker for the disease (Chen Y. et al., 2019). Furthermore, miR-27a-3p in serum EVs in ALS patients is downregulated when compared with normal serum EVs, suggesting that EVs miR-27a-3p could be a detection marker for ALS (Xu et al., 2018).

**TABLE 1:** The potential biomarkers of ALS.

Exosomal source	Species	Alteration markers in ALS patients	ALS biological markers	References
Cerebrospinal fluid	Human	CUE domain-containing 2 (CUEDC2) and Ras-related protein Rab-11A (RAB11A) are highly expressed in cerebrospinal fluid exosomes of ALS patients	CUEDC2 RAB11A	Otake et al., 2019
Blood plasma	Human	miR-146a-5p in exosomes may lead to the loss of motor neurons, and the upregulation of miR-199a-3p affects neuronal regeneration	miR-146a-5p miR-199a-3p	Banack et al., 2020
Blood plasma	Human	The TDP-43 level in plasma exosomes of ALS patients is altered, and the NfL is related to the progression of ALS disease	TDP-43 NfL	Chen P. C. et al., 2020
Serum	Human	Compared with the healthy group, miR-27a-3p level in serum exosomes of ALS patients is decreased	miR-27a-3p	Xu et al., 2018
Central nervous system	Mouse	The presence of misfolded SOD1 in CNS derived exosomes in SOD1^*G*93*A*^ ALS mouse model can instigate ALS	SOD1	Silverman et al., 2019
Astrocyte	Human	IL-6 expression in astrocytic exosomes is increased in ALS patients	IL-6	Chen Y. et al., 2019
Spinal cord neuron	Mouse	miR-124-3p secreted by spinal cord neurons is increased in patients with advanced ALS	miR-124-3p	Yelick et al., 2020
Motor cortex	Human	Proteomics showed the contents of the vascular cellular adhesion molecule-1 (VCAM-1), Endoglin and Ras-related protein R-Ras (RRAS) to be downregulated in ALS	VCAM-1 Endoglin RRAS	Vassileff et al., 2020
Cerebrospinal fluid	Human	The proteasome in the cerebrospinal fluid of ALS patients is decreased, while bleomycin hydrolase expression is downregulated	Proteasome bleomycin hydrolase	Thompson et al., 2020

The biological functions of MSCs EVs have been reported in several studies. Proteomic analysis of MSCs EVs did show most of them to contain proteins related to cell adhesion and apoptotic regulation, as well as proteins related to inflammatory response and myelination (Bonafede et al., 2019; Li et al., 2019). Noteworthy is that MSCs EVs could enhance cell viability by promoting the phosphorylation of AKT and ERK in periodontal ligament cells, and induce cell migration and proliferation (Chew et al., 2019). EVs have dose-dependent characteristics (Arslan et al., 2013; Chew et al., 2019), and can locate the damaged site. Notably, MSCs EVs labeled with gold nanoparticles evidenced their capabilities of crossing the blood-brain barrier (BBB) and migrating to the injured area under the influence of chemokines (Guo et al., 2019).

Extracellular vesicles secreted by ADSCs can promote the differentiation of M1 macrophages into M2 phenotype through miR-21 and CSF-1R, and improve vascular regeneration. Furthermore, ADSCs can secrete more EVs in hypoxic environment, which can strengthen its neuroprotective effect (Zhu D. et al., 2020). Also, ADSCs EVs can transfer miR-25-3p into target cells, and interfere with p53/BNIP3 activation, which can inhibit autophagy and reduce neuronal death (Kuang et al., 2020). This can significantly improve the nerve injury induced by excessive autophagy and promote the recovery of nerve function. Neurite growth is closely related with the formation of synapses. In Alzheimer’s disease, the atrophy of neurites can cause neural dysfunction. A study showed that ADSCs EVs can promote the growth of neurites and participate in synaptic formation (Lee et al., 2018). Additionally, ADSCs EVs can increase the expression of Bcl-2 and reverse neuronal apoptosis. In intranasal administration, EVs combined with gold nanoparticle entered the brain region through the olfactory bulb, and inflammation triggered EVs to migrate to neurons in the injured area (Perets et al., 2019). Because EVs can pass through the BBB and migrate to the damaged site, they have significant protective effects on nerves. The overexpression of SOD1 mutant in motor neuron-like cell line NSC-34 caused oxidative damage to neurons in the H_2_O_2_ environment, though ADSCs EVs treatment reversed the cell damage in an anti-dose-dependent manner (Bonafede et al., 2016). This was in contrast to the study by Chew et al. (2019). Moreover, ADSCs EVs can increase the cell viability of motoneuron like NSC-34 cells transfected with ALS mutant SOD1 and reduce the excessive apoptosis caused by oxidative damage. Also, the ribonuclease RNase 4 contained in ADSCs EVs can play a neuroprotective role (Bonafede et al., 2019). EVs can transfer microRNA from BMSCs to target cells. EVs released by BMSCs, which are perfused with IFN-γ may significantly inhibit immune response (Giunti et al., 2021). Moreover, EVs can reduce the aggregations of TDP-43, SOD1, and FUS. In particular, ADSCs EVs treatment markedly improved the accumulation of SOD1 protein in G93A neurons. In addition, ADSCs EVs prevented the decrements of p-CREB and PGC-1α contents in cells, leading to enhanced conduction of the p-CREB-PGC-1α signaling pathway and mitochondrial function (Lee et al., 2016). Another study showed ADSCs EVs to improve the activity of mitochondrial complex I and membrane potential. Normal SOD1 in ADSCs EVs could neutralize the effect of mutant SOD1, reduce neuronal damage caused by oxidative stress, and exert neuroprotective effects (Calabria et al., 2019).

## The Combination of Adipose-Derived Mesenchymal Stem Cells and Extracellular Vesicles in Improving Amyotrophic Lateral Sclerosis

The global prevalence of ALS is 4.42 per 100,000 people, and the incidence rate appears to be increasing annually (Xu L. et al., 2020). Owing to the increasing burden of ALS, the search for a more effective treatment to delay and improve its development has become imperative in recent times. Both ADSCs and EVs have been suggested as potential interventions for nervous system diseases. However, the bioavailability of EVs seems limited. Chew et al. (2019) showed that injected EVs are considerably reduced after 48 h, which might be due to the degradation of EVs or phagocytosis by cells, or the destruction of the structural integrity of EVs. Transplanted ADSCs may be influenced by the microenvironment, such as local ischemia and hypoxia, oxidative stress and nutritional deficiency that could lead to loss of their therapeutic effects (Shende and Gandhewar, 2020). Also, most of the transplanted cells might die within 1 week after injection, implying that the therapeutic effect of stem cell transplantation alone may be limited (Zhao et al., 2019). The above studies show that the survival status of EVs or ADSCs after injection could be affected by the immune and microenvironment in the body, and hence the therapeutic efficacy of the sole use of either EVs or ADSCs may be limited. Therefore, the combination of ADSCs and EVs could present an effective way to improve the local microenvironment and minimize their loss after injection.

**FIGURE 2:**
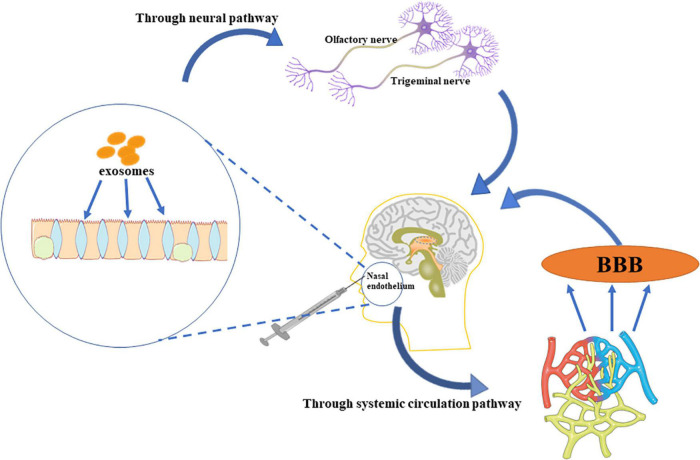
The administration of EVs. EVs delivered intranasally bypass the BBB, and enter the brain directly through the trigeminal and olfactory nerves. Further, some EVs enter the systemic circulation, and permeates the brain through the BBB.

Intravenous and intranasal administration are common methods of delivering EVs. A minimal dose of EVs intravenously administered crosses the BBB to the brain, although the efficiency is not as great as expected (Tian et al., 2018). However, a study showed that EVs enter the brain through the olfactory bulb in the nose (Perets et al., 2019). Some of the EVs via the intranasal route pass through the neural channel formed by olfactory ensheathing cells, bypass the BBB to reach the cerebrospinal fluid, and resulting in EVs distribution in the brain. Also, some EVs pass through the systemic circulation, through the BBB to reach the target area (Agrawal et al., 2018; Bahadur et al., 2020; Hayes et al., 2021; Figure 2).

Different transplantation methods have different effects on neurodegenerative diseases (Table 2). Intrathecal injection of ADSCs expresses higher levels of C-X-C chemokine receptor type 4 (CXCR4). Interestingly, CXCR4 can bind to stromal cell-derived factor-1α (SDF-1α) that are expressed at the injured site, and promote the migration of ADSCs to the damaged site (Ji et al., 2020). Furthermore, ADSCs can relieve neuropathic pain (Jwa et al., 2020). Noteworthy is that intravenous injection does not enhance the passage of ADSCs through the BBB. Although intrathecal injection of ADSCs can pass through the BBB, factors such as gravity and normal circulation of the cerebrospinal fluid can prevent the ADSCs from entering the deep part of brain parenchyma, which will then intricate the required therapeutic concentration of the stem cells (Duma et al., 2019). With most of the ADSCs ending up in the blood vessels of the lungs (Harting et al., 2009), the proposal of a new strategy (i.e., the intracerebroventricular injection) has been suggested (Duma et al., 2019). Although intracerebroventricular injection could impair the blood-brain barrier, it may enhance the conveyance of ADSCs into the brain parenchyma, and thus, increase its level for the patients’ benefit. Also, by protecting motor neurons and secreting neurotrophic factors, the repeated injection of MSCs effectively prolonged the life span of ALS mice and reduced the deterioration of motor function when compared with single injection (Magota et al., 2021). Therefore, in ADSCs treatment, multiple administration may be more effective in improving the pathological degree of ALS.

**TABLE 2:** The different injection methods of ADSCs to ameliorating neurodegenerative diseases.

Diseases	Route of administration	Species	Results	References
Traumatic brain injury	Intravenous injection	Rat	ADSCs regulate inflammation and improve surrounding environment in early stages of trauma	Ruppert et al., 2020
Spinal cord injury	Intrathecal injection	Human	ADSCs injection improve the neurological function of patients, along with continuous recovery in 2 months	Hur et al., 2016
Spinal cord injury	Intravenous injection	Mouse	Most ADSCs remain in the spleen and thymus, with a small amount entering the spinal cord. Also, high dose injection does not cause tumor	Ra et al., 2011
Alzheimer’s disease	Intravenous injection	Rat	ADSCs can migrate to brain tissue and improve learning and memory functions, and melatonin can significantly increase ADSCs effect	Nasiri et al., 2019
Alzheimer’s disease	Intracerebral injection	Mouse	Intravenous injection reduces amyloid deposition and promotes synaptic stability	Chang et al., 2014
Parkinson’s disease	Intracerebral injection	Mouse	Transplanted ADSCs can secrete GDNF to promote their survival and differentiation. However, ADSCs activity decreases with time	Sun S. et al., 2020
Parkinson’s disease	Intravenous injection	Mouse	ADSCs transplantation upregulates the expressions of GDNF and BDNF, leading to the enhanced survival of dopaminergic neurons. More importantly, repeated injection can enhance these effects	Park and Chang, 2020
ALS	Intrathecal injection	Human	High-dose injections are tolerable and safe, but may cause pain	Staff et al., 2016
ALS	Intravenous injection	Human	Intravenous injection of ADSCs prolongs the life of patients and improves ALS symptoms	Shigematsu et al., 2021

MiR-21–encompassed ADSCs EVs could upregulate SDF-1α expression in surrounding cells (An et al., 2019), and EVs derived from ADSCs overexpressing Sirtuin 1 may promote SDF-1α expression in endothelial progenitor cells (Huang H. et al., 2020). Besides, SDF-1α has a significant inducing effect, which can enhance the migration of ADSCs to the damaged area. The involvement of ADSCs in tissue remodeling has been showed recently (Zheng et al., 2020). Motor neurons within the spinal cord expresses higher SDF-1α protein, also known as C-X-C motif chemokine ligand 12 (CXCL12). Intriguingly, the level of CXCL12 increases during the progression of ALS, though the increased content is small. Hence, it cannot play a strong inducing role, and can only be used as a supplementary diagnostic biomarker of ALS (Andrés-Benito et al., 2020). BMSCs expresses higher CXCR4 levels after hypoxia treatment. Therefore, the upregulation of CXCL12 expression in injured neurons may induce MSCs to reach the injured site of motor neurons. Besides, activating the CXCL12/CXCR4 pathway could promote neuronal repair and regeneration (Hu et al., 2019). Hence, by injecting ADSCs EVs, cells can express more SDF-1α (CXCL12). Upregulated expression of SDF-1α can enhance ADSCs homing effect, which can cause its migration to the damaged area.

The combined treatment of ADSCs and EVs has been applied in several diseases. A study showed that the combined treatment of ADSCs and EVs significantly improved the neurological damage after ischemic stroke (Chen et al., 2016). In addition, both anti-inflammatory and antioxidant stress effects formed following the combined treatment was better than the single treatment (Chen et al., 2016). In a mouse skin defect model, the combined treatment of ADSCs and EVs considerably enhanced angiogenesis, inhibited scar, and promoted wound healing. More importantly, the combined treatment of intravenous injection of ADSCs and EVs was better than the single therapy (Zhou et al., 2021). In other studies, the combination of ADSCs and EVs mitigated kidney injury after ischemia-reperfusion and safeguarded the functional and structural integrity of the kidney (Lin et al., 2016), while also playing a protective role by decreasing myocardial infarction insult (Wang et al., 2020). On the basis of the outcomes from the above investigations, we believe that ADSCs combined with EVs could be a potential intervention for the treatment of ALS. However, the advantages and disadvantages of the different injection methods need further investigations. Also, strategies related to enhancing the target effects following the combined use of EVs and ADSCs need to be explored.

Clinically, intrathecal injection of ADSCs is relatively non-invasive, and can alleviate the degree of spinal cord injury and improve nerves (Bydon et al., 2020). Far more, the safety of intrathecal injection of ADSCs has been documented, with the absence of tumorigenicity being reported (Staff et al., 2016). ADSCs come from a wide range of sources and are less difficult to extract, thus, the cost involved in acquiring ADSCs is low (Harasymiak-Krzyżanowska et al., 2013). Intrathecal injection of ADSCs may improve the living environment of ALS motor neurons. This mitigates degeneration and motor neuronal death (Ciervo et al., 2021). Also, intracerebroventricular injection can improve the amount of ADSCs that enters the injured brain injury site of ALS patients, with its safety having been reported in a clinical study (Duma et al., 2019). Interestingly, multiple infusion can significantly minimize dyskinesia in ALS. A study showed CD36 could be used as a specific marker of ADSCs, and both CD271 and CD273 may be used to distinguish BMSCs, which is conducive to the standardization of clinical ADSCs production and ensure patients’ safety (Camilleri et al., 2016). However, long-term culture may reduce the differentiation and nutritional activity of ADSCs, thus, necessitating the need for future investigations to address this problem. Also, a study showed that ADSCs can be separated from the culture medium and survive in normal saline for at least 3 days (Ra et al., 2011), further illustrating the feasibility of ADSCs in clinical treatment. In early clinical trials, the application of MSCs EVs inhibited inflammation and promoted the integrity of lung barrier. However, more cells were needed to produce EVs, making the treatment cost of EVs being relatively high (Liu et al., 2020). Yang et al. (2020) reviewed the available methods for EVs separation. This is paramount to the development of efficient EVs separation technology, which may reduce the cost and contribute to the clinical application of EVs on a global scale (Yang et al., 2020).

In summary, the combined usage of ADSCs EVs and ADSCs may mitigate the degree of motor neuronal degeneration and delay ALS progression.

## Conclusion

A number of studies have evidenced that ADSCs and ADSCs EVs can protect and repair nerve injury in ALS. Moreover, clinical trials have shown that different delivery methods may promote ADSCs and EVs to enter the brain and spinal cord injured regions. ADSCs EVs can enhance the survival and proliferation of ADSCs. Furthermore, ADSCs EVs can form chemokines to induce the migration of ADSCs. Although some amounts of ADSCs and EVs are lost after delivery, the usage of combined therapy via multiple injection methods as well as repeated injections could counteract this problem. ADSCs are easy to obtain and can be used in the treatment of autologous and allogeneic transplantation. Besides, in hypoxia conditions, cells may secrete more EVs. Through *in vitro* isolation and modification, EVs have stronger effects. Therefore, the combinational employment of ADSCs and ADSCs EVs may be a potential treatment for ALS.

## Author Contributions

XL designed the study. XW, YZ, TJ, BB, RF, LW, and XL prepared the first draft of the manuscript and revised the manuscript. All authors approved the final manuscript.

## Conflict of Interest

The authors declare that the research was conducted in the absence of any commercial or financial relationships that could be construed as a potential conflict of interest.

## Publisher’s Note

All claims expressed in this article are solely those of the authors and do not necessarily represent those of their affiliated organizations, or those of the publisher, the editors and the reviewers. Any product that may be evaluated in this article, or claim that may be made by its manufacturer, is not guaranteed or endorsed by the publisher.

## Abbreviations

ADSCs: Adipose-derived mesenchymal stem cells
ALS: Amyotrophic lateral sclerosis
NfL: Neurofilament Light chain protein
TDP-43: TAR DNA binding protein 43
MSCs: Mesenchymal stem cells
EVs: Extracellular Vesicles
BMSCs: Bone marrow mesenchymal stem cells
GDNF: Glial cell-derived neurotrophic factor
BDNF: Brain-derived neurotrophic factor
IL-1 β: Interleukin-1 β
TrkB: Tyrosine receptor kinase B
TNF: tumor necrosis factor
ROS: Reactive oxygen species
Nrf2: Nuclear factor erythroid 2-related factor 2
Keap1: Kelch-like ECH-associated protein 1
ARE: Antioxidant response element
GJA1: Gap junction alpha 1
BBB: Blood-brain barrier
CXCR4: C-X-C chemokine receptor type 4
SDF-1 α: Stromal cell-derived factor-1 α
CXCL12: C-X-C motif chemokine ligand 12
CUEDC2: CUE domain-containing 2
VCAM-1: The vascular cellular adhesion molecule-1
RRAS: Ras-related protein R-Ras
RAB11A: Ras-related protein Rab-11A.

## References

[B1] Agrawal M. Saraf S. Saraf S. Antimisiaris S. G. Chougule M. B. Shoyele S. A. (2018). Nose-to-brain drug delivery: an update on clinical challenges and progress towards approval of anti-Alzheimer drugs. *J. Control. Release.* 281 139–177. 10.1016/j.jconrel.2018.05.011 29772289

[B2] An Y. Zhao J. Nie F. Qin Z. Xue H. Wang G. (2019). Exosomes from adipose-derived stem cells (ADSCs) overexpressing miR-21 promote vascularization of endothelial cells. *Sci. Rep.* 9:12861. 10.1038/s41598-019-49339-y 31492946 PMC6731308

[B3] Andrés-Benito P. Povedano M. Domínguez R. Marco C. Colomina M. J. López-Pérez Ó (2020). Increased C-X-C motif chemokine ligand 12 levels in cerebrospinal fluid as a candidate biomarker in sporadic amyotrophic lateral sclerosis. *Int. J. Mol. Sci.* 21:8680. 10.3390/ijms21228680 33213069 PMC7698527

[B4] Angeloni C. Gatti M. Prata C. Hrelia S. Maraldi T. (2020). Role of mesenchymal stem cells in counteracting oxidative stress-related neurodegeneration. *Int. J. Mol. Sci.* 21:3299. 10.3390/ijms21093299 32392722 PMC7246730

[B5] Arslan F. Lai R. C. Smeets M. B. Akeroyd L. Choo A. Aguor E. N. (2013). Mesenchymal stem cell-derived exosomes increase ATP levels, decrease oxidative stress and activate PI3K/Akt pathway to enhance myocardial viability and prevent adverse remodeling after myocardial ischemia/reperfusion injury. *Stem Cell Res.* 10 301–312. 10.1016/j.scr.2013.01.002 23399448

[B6] Bahadur S. Sachan N. Harwansh R. K. Deshmukh R. (2020). Nanoparticlized system: promising approach for the management of Alzheimer’s disease through intranasal delivery. *Curr. Pharm. Des.* 26 1331–1344. 10.2174/1381612826666200311131658 32160843

[B7] Banack S. A. Dunlop R. A. Cox P. A. (2020). An miRNA fingerprint using neural-enriched extracellular vesicles from blood plasma: towards a biomarker for amyotrophic lateral sclerosis/motor neuron disease. *Open Biol.* 10:200116. 10.1098/rsob.200116 32574550 PMC7333885

[B8] Blokhuis A. M. Groen E. J. Koppers M. van den Berg L. H. Pasterkamp R. J. (2013). Protein aggregation in amyotrophic lateral sclerosis. *Acta Neuropathol.* 125 777–794. 10.1007/s00401-013-1125-6 23673820 PMC3661910

[B9] Bonafede R. Brandi J. Manfredi M. Scambi I. Schiaffino L. Merigo F. (2019). The anti-apoptotic effect of ASC-exosomes in an in vitro ALS model and their proteomic analysis. *Cells* 8:1087. 10.3390/cells8091087 31540100 PMC6770878

[B10] Bonafede R. Scambi I. Peroni D. Potrich V. Boschi F. Benati D. (2016). Exosome derived from murine adipose-derived stromal cells: neuroprotective effect on in vitro model of amyotrophic lateral sclerosis. *Exp. Cell Res.* 340 150–158. 10.1016/j.yexcr.2015.12.009 26708289

[B11] Bonifacino T. Rebosio C. Provenzano F. Torazza C. Balbi M. Milanese M. (2019). Enhanced function and overexpression of metabotropic glutamate receptors 1 and 5 in the spinal cord of the SOD1G93A mouse model of amyotrophic lateral sclerosis during disease progression. *Int. J. Mol. Sci.* 20:4552. 10.3390/ijms20184552 31540330 PMC6774337

[B12] Bydon M. Dietz A. B. Goncalves S. Moinuddin F. M. Alvi M. A. Goyal A. (2020). CELLTOP clinical trial: first report from a phase 1 trial of autologous adipose tissue-derived mesenchymal stem cells in the treatment of paralysis due to traumatic spinal cord injury. *Mayo Clin. Proc.* 95 406–414. 10.1016/j.mayocp.2019.10.008 31785831

[B13] Calabria E. Scambi I. Bonafede R. Schiaffino L. Peroni D. Potrich V. (2019). ASCs-exosomes recover coupling efficiency and mitochondrial membrane potential in an in vitro model of ALS. *Front. Neurosci.* 13:1070. 10.3389/fnins.2019.01070 31680811 PMC6811497

[B14] Camilleri E. T. Gustafson M. P. Dudakovic A. Riester S. M. Garces C. G. Paradise C. R. (2016). Identification and validation of multiple cell surface markers of clinical-grade adipose-derived mesenchymal stromal cells as novel release criteria for good manufacturing practice-compliant production. *Stem Cell Res. Ther.* 7:107. 10.1186/s13287-016-0370-8 27515308 PMC4982273

[B15] Chang K. A. Kim H. J. Joo Y. Ha S. Suh Y. H. (2014). The therapeutic effects of human adipose-derived stem cells in Alzheimer’s disease mouse models. *Neurodegener. Dis.* 13 99–102. 10.1159/000355261 24157626

[B16] Chen K. H. Chen C. H. Wallace C. G. Yuen C. M. Kao G. S. Chen Y. L. (2016). Intravenous administration of xenogenic adipose-derived mesenchymal stem cells (ADMSC) and ADMSC-derived exosomes markedly reduced brain infarct volume and preserved neurological function in rat after acute ischemic stroke. *Oncotarget* 7 74537–74556. 10.18632/oncotarget.12902 27793019 PMC5342685

[B17] Chen P. C. Wu D. Hu C. J. Chen H. Y. Hsieh Y. C. Huang C. C. (2020). Exosomal TAR DNA-binding protein-43 and neurofilaments in plasma of amyotrophic lateral sclerosis patients: a longitudinal follow-up study. *J. Neurol. Sci.* 418:117070. 10.1016/j.jns.2020.117070 32836016

[B18] Chen W. Zheng P. Hong T. Wang Y. Liu N. He B. (2020). Astrocytes-derived exosomes induce neuronal recovery after traumatic brain injury via delivering gap junction alpha 1-20 k. *J. Tissue Eng. Regen. Med.* 14 412–423. 10.1002/term.3002 31826322

[B19] Chen Y. Xia K. Chen L. Fan D. (2019). Increased Interleukin-6 levels in the astrocyte-derived exosomes of sporadic amyotrophic lateral sclerosis patients. *Front. Neurosci.* 13:574. 10.3389/fnins.2019.00574 31231184 PMC6560167

[B20] Chen Z. Zhong H. Wei J. Lin S. Zong Z. Gong F. (2019). Inhibition of Nrf2/HO-1 signaling leads to increased activation of the NLRP3 inflammasome in osteoarthritis. *Arthritis Res. Ther.* 21:300. 10.1186/s13075-019-2085-6 31870428 PMC6929452

[B21] Chew J. R. J. Chuah S. J. Teo K. Y. W. Zhang S. Lai R. C. Fu J. H. (2019). Mesenchymal stem cell exosomes enhance periodontal ligament cell functions and promote periodontal regeneration. *Acta Biomater.* 89 252–264. 10.1016/j.actbio.2019.03.021 30878447

[B22] Chiò A. Mazzini L. Mora G. (2020). Disease-modifying therapies in amyotrophic lateral sclerosis. *Neuropharmacology* 167:107986. 10.1016/j.neuropharm.2020.107986 32062193

[B23] Ciervo Y. Gatto N. Allen C. Grierson A. Ferraiuolo L. Mead R. J. (2021). Adipose-derived stem cells protect motor neurons and reduce glial activation in both in vitro and in vivo models of ALS. *Mol. Ther. Methods Clin. Dev.* 21 413–433. 10.1016/j.omtm.2021.03.017 33869658 PMC8044387

[B24] Constantin G. Marconi S. Rossi B. Angiari S. Calderan L. Anghileri E. (2009). Adipose-derived mesenchymal stem cells ameliorate chronic experimental autoimmune encephalomyelitis. *Stem Cells* 27 2624–2635. 10.1002/stem.194 19676124

[B25] Deng Z. Lim J. Wang Q. Purtell K. Wu S. Palomo G. M. (2020). ALS-FTLD-linked mutations of SQSTM1/p62 disrupt selective autophagy and NFE2L2/NRF2 anti-oxidative stress pathway. *Autophagy* 16 917–931. 10.1080/15548627.2019.1644076 31362587 PMC7144840

[B26] Deora V. Lee J. D. Albornoz E. A. McAlary L. Jagaraj C. J. Robertson A. A. B. (2020). The microglial NLRP3 inflammasome is activated by amyotrophic lateral sclerosis proteins. *Glia* 68 407–421. 10.1002/glia.23728 31596526

[B27] Ding H. Chen J. Su M. Lin Z. Zhan H. Yang F. (2020). BDNF promotes activation of astrocytes and microglia contributing to neuroinflammation and mechanical allodynia in cyclophosphamide-induced cystitis. *J. Neuroinflammation* 17:19. 10.1186/s12974-020-1704-0 31931832 PMC6958761

[B28] Doyle L. M. Wang M. Z. (2019). Overview of extracellular vesicles, their origin, composition, purpose, and methods for exosome isolation and analysis. *Cells* 8:727. 10.3390/cells8070727 31311206 PMC6678302

[B29] Duma C. Kopyov O. Kopyov A. Berman M. Lander E. Elam M. (2019). Human intracerebroventricular (ICV) injection of autologous, non-engineered, adipose-derived stromal vascular fraction (ADSVF) for neurodegenerative disorders: results of a 3-year phase 1 study of 113 injections in 31 patients. *Mol. Biol. Rep.* 46 5257–5272. 10.1007/s11033-019-04983-5 31327120

[B30] Elsharkawi F. Elsabah M. Shabayek M. Khaled H. (2019). Urine and serum exosomes as novel biomarkers in detection of bladder cancer. *Asian Pac. J. Cancer Prev.* 20 2219–2224. 10.31557/APJCP.2019.20.7.2219 31350988 PMC6745236

[B31] Frühbeis C. Fröhlich D. Kuo W. P. Amphornrat J. Thilemann S. Saab A. S. (2013). Neurotransmitter-triggered transfer of exosomes mediates oligodendrocyte-neuron communication. *PLoS Biol.* 11:e1001604. 10.1371/journal.pbio.1001604 23874151 PMC3706306

[B32] Furue M. (2020). Regulation of filaggrin, loricrin, and involucrin by IL-4, IL-13, IL-17A, IL-22, AHR, and NRF2: pathogenic implications in atopic dermatitis. *Int. J. Mol. Sci.* 21:5382. 10.3390/ijms21155382 32751111 PMC7432778

[B33] Gao S. Guo X. Zhao S. Jin Y. Zhou F. Yuan P. (2019). Differentiation of human adipose-derived stem cells into neuron/motoneuron-like cells for cell replacement therapy of spinal cord injury. *Cell Death Dis.* 10:597. 10.1038/s41419-019-1772-1 31395857 PMC6687731

[B34] Garrido-Pascual P. Alonso-Varona A. Castro B. Burón M. Palomares T. (2020). H_2_O_2_-preconditioned human adipose-derived stem cells (HC016) increase their resistance to oxidative stress by overexpressing Nrf2 and bioenergetic adaptation. *Stem Cell Res. Ther.* 11:335. 10.1186/s13287-020-01851-z 32746890 PMC7397657

[B35] Geraghty A. C. Gibson E. M. Ghanem R. A. Greene J. J. Ocampo A. Goldstein A. K. (2019). Loss of adaptive myelination contributes to methotrexate chemotherapy-related cognitive impairment. *Neuron* 103 250–265.e8. 10.1016/j.neuron.2019.04.032 31122677 PMC6697075

[B36] Giunti D. Marini C. Parodi B. Usai C. Milanese M. Bonanno G. (2021). Role of miRNAs shuttled by mesenchymal stem cell-derived small extracellular vesicles in modulating neuroinflammation. *Sci. Rep.* 11:1740. 10.1038/s41598-021-81039-4 33462263 PMC7814007

[B37] Goedee H. S. Sleutjes B. T. H. M. van Es M. A. van den Berg L. H. (2020). Neuro-imaging in amyotrophic lateral sclerosis: should we shift towards the periphery? *Clin. Neurophysiol.* 131 2286–2288. 10.1016/j.clinph.2020.06.004 32622587

[B38] Gonçalves N. P. Yan Y. Ulrichsen M. Venø M. T. Poulsen E. T. Enghild J. J. (2020). Modulation of small RNA signatures in Schwann-cell-derived extracellular vesicles by the p75 neurotrophin receptor and sortilin. *Biomedicines* 8:450. 10.3390/biomedicines8110450 33114403 PMC7694014

[B39] Gugliandolo A. Bramanti P. Mazzon E. (2019). Mesenchymal stem cells: a potential therapeutic approach for amyotrophic lateral sclerosis? *Stem Cells Int.* 2019:3675627. 10.1155/2019/3675627 30956667 PMC6431432

[B40] Guo S. Perets N. Betzer O. Ben-Shaul S. Sheinin A. Michaelevski I. (2019). Intranasal delivery of mesenchymal stem cell derived exosomes loaded with phosphatase and tensin homolog siRNA repairs complete spinal cord injury. *ACS Nano* 13 10015–10028. 10.1021/acsnano.9b01892 31454225

[B41] Harasymiak-Krzyżanowska I. Niedojadło A. Karwat J. Kotuła L. Gil-Kulik P. Sawiuk M. (2013). Adipose tissue-derived stem cells show considerable promise for regenerative medicine applications. *Cell. Mol. Biol. Lett.* 18 479–493. 10.2478/s11658-013-0101-4 23949841 PMC6275722

[B42] Hardiman O. (2021). Major advances in amyotrophic lateral sclerosis in 2020. *Lancet Neurol.* 20 14–15. 10.1016/S1474-4422(20)30447-6 33340474

[B43] Harting M. T. Jimenez F. Xue H. Fischer U. M. Baumgartner J. Dash P. K. (2009). Intravenous mesenchymal stem cell therapy for traumatic brain injury. *J. Neurosurg.* 110 1189–1197. 10.3171/2008.9.JNS08158 19301973 PMC2889620

[B44] Hayes S. H. Liu Q. Selvakumaran S. Haney M. J. Batrakova E. V. Allman B. L. (2021). Brain targeting and toxicological assessment of the extracellular vesicle-packaged antioxidant catalase-SKL following intranasal administration in mice. *Neurotox. Res.* 39 1418–1429. 10.1007/s12640-021-00390-6 34196954

[B45] He C. Zheng S. Luo Y. Wang B. (2018). Exosome theranostics: biology and translational medicine. *Theranostics* 8 237–255. 10.7150/thno.21945 29290805 PMC5743472

[B46] Hsu M. N. Liao H. T. Truong V. A. Huang K. L. Yu F. J. Chen H. H. (2019). CRISPR-based activation of endogenous neurotrophic genes in adipose stem cell sheets to stimulate peripheral nerve regeneration. *Theranostics* 9 6099–6111. 10.7150/thno.36790 31534539 PMC6735509

[B47] Hu Y. Cao C. Qin X. Y. Yu Y. Yuan J. Zhao Y. (2017). Increased peripheral blood inflammatory cytokine levels in amyotrophic lateral sclerosis: a meta-analysis study. *Sci. Rep.* 7:9094. 10.1038/s41598-017-09097-1 28831083 PMC5567306

[B48] Hu Y. Chen W. Wu L. Jiang L. Qin H. Tang N. (2019). Hypoxic preconditioning improves the survival and neural effects of transplanted mesenchymal stem cells via CXCL12/CXCR4 signalling in a rat model of cerebral infarction. *Cell Biochem. Funct.* 37 504–515. 10.1002/cbf.3423 31368195

[B49] Hu Y. Liu S. Zhu B. M. (2020). CRISPR/Cas9-induced loss of Keap1 enhances anti-oxidation in rat adipose-derived mesenchymal stem cells. *Front. Neurol.* 10:1311. 10.3389/fneur.2019.01311 32132961 PMC7040357

[B50] Huang H. Xu Z. Qi Y. Zhang W. Zhang C. Jiang M. (2020). Exosomes from SIRT1-overexpressing ADSCs restore cardiac function by improving angiogenic function of EPCs. *Mol. Ther. Nucleic Acids* 21 737–750. 10.1016/j.omtn.2020.07.007 32771925 PMC7412761

[B51] Huang X. Fei G. Q. Liu W. J. Ding J. Wang Y. Wang H. (2020). Adipose-derived mesenchymal stem cells protect against CMS-induced depression-like behaviors in mice via regulating the Nrf2/HO-1 and TLR4/NF-κB signaling pathways. *Acta Pharmacol. Sin.* 41 612–619. 10.1038/s41401-019-0317-6 31796867 PMC7468309

[B52] Hur J. W. Cho T. H. Park D. H. Lee J. B. Park J. Y. Chung Y. G. (2016). Intrathecal transplantation of autologous adipose-derived mesenchymal stem cells for treating spinal cord injury: a human trial. *J. Spinal Cord Med.* 39 655–664. 10.1179/2045772315Y.0000000048 26208177 PMC5137573

[B53] Jain G. Stuendl A. Rao P. Berulava T. Pena Centeno T. Kaurani L. (2019). A combined miRNA-piRNA signature to detect Alzheimer’s disease. *Transl. Psychiatry* 9:250. 10.1038/s41398-019-0579-2 31591382 PMC6779890

[B54] Ji F. Wang Y. Yuan J. Wu Q. Wang J. Liu D. (2020). The potential role of stromal cell-derived factor-1α/CXCR4/CXCR7 axis in adipose-derived mesenchymal stem cells. *J. Cell. Physiol.* 235 3548–3557. 10.1002/jcp.29243 31566725 PMC13482690

[B55] Johnsen B. (2020). Diagnostic criteria for amyotrophic lateral sclerosis from El Escorial to Gold Coast. *Clin. Neurophysiol.* 131 1962–1963. 10.1016/j.clinph.2020.04.012 32418823

[B56] Joshi A. U. Minhas P. S. Liddelow S. A. Haileselassie B. Andreasson K. I. Dorn G. W. II (2019). Fragmented mitochondria released from microglia trigger A1 astrocytic response and propagate inflammatory neurodegeneration. *Nat. Neurosci.* 22 1635–1648. 10.1038/s41593-019-0486-0 31551592 PMC6764589

[B57] Just-Borràs L. Hurtado E. Cilleros-Mañé V. Biondi O. Charbonnier F. Tomàs M. (2019). Overview of impaired BDNF signaling, their coupled downstream serine-threonine kinases and SNARE/SM complex in the neuromuscular junction of the amyotrophic lateral sclerosis model SOD1-G93A mice. *Mol. Neurobiol.* 56 6856–6872. 10.1007/s12035-019-1550-1 30929165

[B58] Jwa H. S. Kim Y. H. Lee J. Back S. K. Park C. K. (2020). Adipose tissue-derived stem cells alleviate cold allodynia in a rat spinal nerve ligation model of neuropathic pain. *Stem Cells Int.* 2020:8845262. 10.1155/2020/8845262 33101421 PMC7576351

[B59] Kalluri R. LeBleu V. S. (2020). The biology, function, and biomedical applications of exosomes. *Science* 367:eaau6977. 10.1126/science.aau6977 32029601 PMC7717626

[B60] Kim K. S. Lee H. J. An J. Kim Y. B. Ra J. C. Lim I. (2014). Transplantation of human adipose tissue-derived stem cells delays clinical onset and prolongs life span in ALS mouse model. *Cell Transplant.* 23 1585–1597. 10.3727/096368913X673450 24070071

[B61] Kingham P. J. Kolar M. K. Novikova L. N. Novikov L. N. Wiberg M. (2014). Stimulating the neurotrophic and angiogenic properties of human adipose-derived stem cells enhances nerve repair. *Stem Cells Dev.* 23 741–754. 10.1089/scd.2013.0396 24124760

[B62] Kuang Y. Zheng X. Zhang L. Ai X. Venkataramani V. Kilic E. (2020). Adipose-derived mesenchymal stem cells reduce autophagy in stroke mice by extracellular vesicle transfer of miR-25. *J. Extracell. Vesicles* 10:e12024. 10.1002/jev2.12024 33304476 PMC7710129

[B63] Lee M. Ban J. J. Kim K. Y. Jeon G. S. Im W. Sung J. J. (2016). Adipose-derived stem cell exosomes alleviate pathology of amyotrophic lateral sclerosis in vitro. *Biochem. Biophys. Res. Commun.* 479 434–439. 10.1016/j.bbrc.2016.09.069 27641665

[B64] Lee M. Ban J. J. Yang S. Im W. Kim M. (2018). The exosome of adipose-derived stem cells reduces β-amyloid pathology and apoptosis of neuronal cells derived from the transgenic mouse model of Alzheimer’s disease. *Brain Res.* 1691 87–93. 10.1016/j.brainres.2018.03.034 29625119

[B65] Li Z. Liu F. He X. Yang X. Shan F. Feng J. (2019). Exosomes derived from mesenchymal stem cells attenuate inflammation and demyelination of the central nervous system in EAE rats by regulating the polarization of microglia. *Int. Immunopharmacol.* 67 268–280. 10.1016/j.intimp.2018.12.001 30572251

[B66] Lin K. C. Yip H. K. Shao P. L. Wu S. C. Chen K. H. Chen Y. T. (2016). Combination of adipose-derived mesenchymal stem cells (ADMSC) and ADMSC-derived exosomes for protecting kidney from acute ischemia-reperfusion injury. *Int. J. Cardiol.* 216 173–185. 10.1016/j.ijcard.2016.04.061 27156061

[B67] Liu A. Zhang X. He H. Zhou L. Naito Y. Sugita S. (2020). Therapeutic potential of mesenchymal stem/stromal cell-derived secretome and vesicles for lung injury and disease. *Expert Opin. Biol. Ther.* 20 125–140. 10.1080/14712598.2020.1689954 31701782 PMC6981051

[B68] Lo Furno D. Mannino G. Giuffrida R. Gili E. Vancheri C. Tarico M. S. (2018). Neural differentiation of human adipose-derived mesenchymal stem cells induced by glial cell conditioned media. *J. Cell. Physiol.* 233 7091–7100. 10.1002/jcp.26632 29737535

[B69] Lyon M. S. Wosiski-Kuhn M. Gillespie R. Caress J. Milligan C. (2019). Inflammation, immunity, and amyotrophic lateral sclerosis: I. etiology and pathology. *Muscle Nerve* 59 10–22. 10.1002/mus.26289 29979464

[B70] Ma H. Lam P. K. Tong C. S. W. Lo K. K. Y. Wong G. K. C. Poon W. S. (2019). The neuroprotection of hypoxic adipose tissue-derived mesenchymal stem cells in experimental traumatic brain injury. *Cell Transplant.* 28 874–884. 10.1177/0963689719855624 31185737 PMC6719502

[B71] Magota H. Sasaki M. Kataoka-Sasaki Y. Oka S. Ukai R. Kiyose R. (2021). Repeated infusion of mesenchymal stem cells maintain the condition to inhibit deteriorated motor function, leading to an extended lifespan in the SOD1G93A rat model of amyotrophic lateral sclerosis. *Mol. Brain* 14:76. 10.1186/s13041-021-00787-6 33962678 PMC8103621

[B72] Marconi S. Bonaconsa M. Scambi I. Squintani G. M. Rui W. Turano E. (2013). Systemic treatment with adipose-derived mesenchymal stem cells ameliorates clinical and pathological features in the amyotrophic lateral sclerosis murine model. *Neuroscience* 248 333–343. 10.1016/j.neuroscience.2013.05.034 23727509

[B73] Marconi S. Castiglione G. Turano E. Bissolotti G. Angiari S. Farinazzo A. (2012). Human adipose-derived mesenchymal stem cells systemically injected promote peripheral nerve regeneration in the mouse model of sciatic crush. *Tissue Eng. Part A* 18 1264–1272. 10.1089/ten.TEA.2011.0491 22332955

[B74] Marote A. Teixeira F. G. Mendes-Pinheiro B. Salgado A. J. (2016). MSCs-derived exosomes: cell-secreted nanovesicles with regenerative potential. *Front. Pharmacol.* 7:231. 10.3389/fphar.2016.00231 27536241 PMC4971062

[B75] Masgutov R. Masgutova G. Mullakhmetova A. Zhuravleva M. Shulman A. Rogozhin A. (2019). Adipose-derived mesenchymal stem cells applied in fibrin glue stimulate peripheral nerve regeneration. *Front. Med.* 6:68. 10.3389/fmed.2019.00068 31024916 PMC6465797

[B76] Masrori P. Van Damme P. (2020). Amyotrophic lateral sclerosis: a clinical review. *Eur. J. Neurol.* 27 1918–1929. 10.1111/ene.14393 32526057 PMC7540334

[B77] McCauley M. E. Baloh R. H. (2019). Inflammation in ALS/FTD pathogenesis. *Acta Neuropathol.* 137 715–730. 10.1007/s00401-018-1933-9 30465257 PMC6482122

[B78] McCauley M. E. O’Rourke J. G. Yáñez A. Markman J. L. Ho R. Wang X. (2020). C9orf72 in myeloid cells suppresses STING-induced inflammation. *Nature* 585 96–101. 10.1038/s41586-020-2625-x 32814898 PMC7484469

[B79] Mi B. Chen L. Xiong Y. Yan C. Xue H. Panayi A. C. (2020). Saliva exosomes-derived UBE2O mRNA promotes angiogenesis in cutaneous wounds by targeting SMAD6. *J. Nanobiotechnol.* 18:68. 10.1186/s12951-020-00624-3 32375794 PMC7203970

[B80] Munguía-Martínez M. F. Nava-Ruíz C. Ruíz-Díaz A. Díaz-Ruíz A. Yescas-Gómez P. Méndez-Armenta M. (2019). Immunohistochemical study of antioxidant enzymes regulated by Nrf2 in the models of epileptic seizures (KA and PTZ). *Oxid. Med. Cell. Longev.* 2019:1327986. 10.1155/2019/1327986 31019649 PMC6451808

[B81] Nasiri E. Alizadeh A. Roushandeh A. M. Gazor R. Hashemi-Firouzi N. Golipoor Z. (2019). Melatonin-pretreated adipose-derived mesenchymal stem cells efficeintly improved learning, memory, and cognition in an animal model of Alzheimer’s disease. *Metab. Brain Dis.* 34 1131–1143. 10.1007/s11011-019-00421-4 31129766

[B82] Niedermeyer S. Murn M. Choi P. J. (2019). Respiratory failure in amyotrophic lateral sclerosis. *Chest* 155 401–408. 10.1016/j.chest.2018.06.035 29990478

[B83] Noman A. S. M. Parag R. R. Rashid M. I. Islam S. Rahman M. Z. Chowdhury A. A. (2020). Chemotherapeutic resistance of head and neck squamous cell carcinoma is mediated by EpCAM induction driven by IL-6/p62 associated Nrf2-antioxidant pathway activation. *Cell Death Dis.* 11:663. 10.1038/s41419-020-02907-x 32814771 PMC7438524

[B84] Ohta Y. Nomura E. Shang J. Feng T. Huang Y. Liu X. (2019). Enhanced oxidative stress and the treatment by edaravone in mice model of amyotrophic lateral sclerosis. *J. Neurosci. Res.* 97 607–619. 10.1002/jnr.24368 30565312

[B85] Otake K. Kamiguchi H. Hirozane Y. (2019). Identification of biomarkers for amyotrophic lateral sclerosis by comprehensive analysis of exosomal mRNAs in human cerebrospinal fluid. *BMC Med. Genomics* 12:7. 10.1186/s12920-019-0473-z 30630471 PMC6329125

[B86] Otsuka T. Maeda Y. Kurose T. Nakagawa K. Mitsuhara T. Kawahara Y. (2021). Comparisons of neurotrophic effects of mesenchymal stem cells derived from different tissues on chronic spinal cord injury rats. *Stem Cells Dev.* 30 865–875. 10.1089/scd.2021.0070 34148410

[B87] Park H. Chang K. A. (2020). Therapeutic potential of repeated intravenous transplantation of human adipose-derived stem cells in subchronic MPTP-induced Parkinson’s disease mouse model. *Int. J. Mol. Sci.* 21:8129. 10.3390/ijms21218129 33143234 PMC7663651

[B88] Paudyal A. Ghinea F. S. Driga M. P. Fang W. H. Alessandri G. Combes L. (2021). p5 Peptide-loaded human adipose-derived mesenchymal stem cells promote neurological recovery after focal cerebral ischemia in a rat model. *Transl. Stroke Res.* 12 125–135. 10.1007/s12975-020-00805-0 32378028 PMC7803698

[B89] Perets N. Betzer O. Shapira R. Brenstein S. Angel A. Sadan T. (2019). Golden exosomes selectively target brain pathologies in neurodegenerative and neurodevelopmental disorders. *Nano Lett.* 19 3422–3431. 10.1021/acs.nanolett.8b04148 30761901

[B90] Phukan J. Pender N. P. Hardiman O. (2007). Cognitive impairment in amyotrophic lateral sclerosis. *Lancet Neurol.* 6 994–1003. 10.1016/S1474-4422(07)70265-X 17945153

[B91] Prpar Mihevc S. Kokondoska Grgich V. Kopitar A. N. Mohoriè L. Majdiè G. (2020). Neural differentiation of canine mesenchymal stem cells/multipotent mesenchymal stromal cells. *BMC Vet. Res.* 16:282. 10.1186/s12917-020-02493-2 32778115 PMC7418429

[B92] Ra J. C. Shin I. S. Kim S. H. Kang S. K. Kang B. C. Lee H. Y. (2011). Safety of intravenous infusion of human adipose tissue-derived mesenchymal stem cells in animals and humans. *Stem Cells Dev.* 20 1297–1308. 10.1089/scd.2010.0466 21303266

[B93] Rahman M. A. Kodidela S. Sinha N. Haque S. Shukla P. K. Rao R. (2019). Plasma exosomes exacerbate alcohol- and acetaminophen-induced toxicity via CYP2E1 pathway. *Sci. Rep.* 9:6571. 10.1038/s41598-019-43064-2 31024054 PMC6484097

[B94] Řehořová M. Vargová I. Forostyak S. Vacková I. Turnovcová K. Kupcová Skalníková H. (2019). A combination of intrathecal and intramuscular application of human mesenchymal stem cells partly reduces the activation of necroptosis in the spinal cord of SOD1G93A Rats. *Stem Cells Transl. Med.* 8 535–547. 10.1002/sctm.18-0223 30802001 PMC6525562

[B95] Ritter A. Friemel A. Roth S. Kreis N. N. Hoock S. C. Safdar B. K. (2019). Subcutaneous and visceral adipose-derived mesenchymal stem cells: commonality and diversity. *Cells* 8:1288. 10.3390/cells8101288 31640218 PMC6830091

[B96] Romano N. Catalani A. Lattante S. Belardo A. Proietti S. Bertini L. (2020). ALS skin fibroblasts reveal oxidative stress and ERK1/2-mediated cytoplasmic localization of TDP-43. *Cell. Signal.* 70:109591. 10.1016/j.cellsig.2020.109591 32126264

[B97] Roszek K. Makowska N. Czarnecka J. Porowińska D. Da̧browski M. Danielewska J. (2017). Canine adipose-derived stem cells: purinergic characterization and neurogenic potential for therapeutic applications. *J. Cell. Biochem.* 118 58–65. 10.1002/jcb.25610 27225588

[B98] Ruppert K. A. Prabhakara K. S. Toledano-Furman N. E. Udtha S. Arceneaux A. Q. Park H. (2020). Human adipose-derived mesenchymal stem cells for acute and sub-acute TBI. *PLoS One* 15:e0233263. 10.1371/journal.pone.0233263 32453741 PMC7250455

[B99] Seredenina T. Nayernia Z. Sorce S. Maghzal G. J. Filippova A. Ling S. C. (2016). Evaluation of NADPH oxidases as drug targets in a mouse model of familial amyotrophic lateral sclerosis. *Free Radic. Biol. Med.* 97 95–108. 10.1016/j.freeradbiomed.2016.05.016 27212019

[B100] Shamadykova D. V. Panteleev D. Y. Kust N. N. Savchenko E. A. Rybalkina E. Y. Revishchin A. V. (2021). Neuroinductive properties of mGDNF depend on the producer, *E. coli* or human cells. *PLoS One* 16:e0258289. 10.1371/journal.pone.0258289 34634077 PMC8504721

[B101] Shende P. Gandhewar N. (2020). Current trend and pro-survival approaches for augmenting stem cell viability. *Curr. Pharm. Biotechnol.* 21 1154–1164. 10.2174/1389201021666200416130253 32297579

[B102] Shigematsu K. Takeda T. Komori N. Urushihata N. Oki K. Tahara K. (2021). Long-term survival of a patient with amyotrophic lateral sclerosis (ALS) who received autologous adipose-derived mesenchymal stem cells. *Eur. Rev. Med. Pharmacol. Sci.* 25 4086–4090. 10.26355/eurrev_202106_26050 34156687

[B103] Silverman J. M. Christy D. Shyu C. C. Moon K. M. Fernando S. Gidden Z. (2019). CNS-derived extracellular vesicles from superoxide dismutase 1 (SOD1)G93A ALS mice originate from astrocytes and neurons and carry misfolded SOD1. *J. Biol. Chem.* 294 3744–3759. 10.1074/jbc.RA118.004825 30635404 PMC6416428

[B104] Song H. Li X. Zhao Z. Qian J. Wang Y. Cui J. (2019). Reversal of osteoporotic activity by endothelial cell-secreted bone targeting and biocompatible exosomes. *Nano Lett.* 19 3040–3048. 10.1021/acs.nanolett.9b00287 30968694

[B105] Song Y. Li Z. He T. Qu M. Jiang L. Li W. (2019). M2 microglia-derived exosomes protect the mouse brain from ischemia-reperfusion injury via exosomal miR-124. *Theranostics* 9 2910–2923. 10.7150/thno.30879 31244932 PMC6568171

[B106] Staff N. P. Madigan N. N. Morris J. Jentoft M. Sorenson E. J. Butler G. (2016). Safety of intrathecal autologous adipose-derived mesenchymal stromal cells in patients with ALS. *Neurology* 87 2230–2234. 10.1212/WNL.0000000000003359 27784774 PMC5123559

[B107] Strioga M. Viswanathan S. Darinskas A. Slaby O. Michalek J. (2012). Same or not the same? Comparison of adipose tissue-derived versus bone marrow-derived mesenchymal stem and stromal cells. *Stem Cells Dev.* 21 2724–2752. 10.1089/scd.2011.0722 22468918

[B108] Sun S. Zhang Q. Li M. Gao P. Huang K. Beejadhursing R. (2020). GDNF promotes survival and therapeutic efficacy of human adipose-derived mesenchymal stem cells in a mouse model of Parkinson’s disease. *Cell Transplant.* 29:963689720908512. 10.1177/0963689720908512 32292068 PMC7444207

[B109] Sun Y. He L. Wang T. Hua W. Qin H. Wang J. (2020). Activation of p62-Keap1-Nrf2 pathway protects 6-hydroxydopamine-induced ferroptosis in dopaminergic cells. *Mol. Neurobiol.* 57 4628–4641. 10.1007/s12035-020-02049-3 32770451

[B110] Sykova E. Cizkova D. Kubinova S. (2021). Mesenchymal stem cells in treatment of spinal cord injury and amyotrophic lateral sclerosis. *Front. Cell Dev. Biol.* 9:695900. 10.3389/fcell.2021.695900 34295897 PMC8290345

[B111] Thompson A. G. Gray E. Mäger I. Thézénas M. L. Charles P. D. Talbot K. (2020). CSF extracellular vesicle proteomics demonstrates altered protein homeostasis in amyotrophic lateral sclerosis. *Clin. Proteomics* 17:31. 10.1186/s12014-020-09294-7 32821252 PMC7433176

[B112] Tian T. Zhang H. X. He C. P. Fan S. Zhu Y. L. Qi C. (2018). Surface functionalized exosomes as targeted drug delivery vehicles for cerebral ischemia therapy. *Biomaterials* 150 137–149. 10.1016/j.biomaterials.2017.10.012 29040874

[B113] Trias E. King P. H. Si Y. Kwon Y. Varela V. Ibarburu S. (2018). Mast cells and neutrophils mediate peripheral motor pathway degeneration in ALS. *JCI Insight* 3:e123249. 10.1172/jci.insight.123249 30282815 PMC6237484

[B114] van Es M. A. Hardiman O. Chio A. Al-Chalabi A. Pasterkamp R. J. Veldink J. H. (2017). Amyotrophic lateral sclerosis. *Lancet* 390 2084–2098. 10.1016/S0140-6736(17)31287-4 28552366

[B115] Vassileff N. Vella L. J. Rajapaksha H. Shambrook M. Kenari A. N. McLean C. (2020). Revealing the proteome of motor cortex derived extracellular vesicles isolated from amyotrophic lateral sclerosis human postmortem tissues. *Cells* 9:1709. 10.3390/cells9071709 32708779 PMC7407138

[B116] Wang P. Deng J. Dong J. Liu J. Bigio E. H. Mesulam M. (2019). TDP-43 induces mitochondrial damage and activates the mitochondrial unfolded protein response. *PLoS Genet.* 15:e1007947. 10.1371/journal.pgen.1007947 31100073 PMC6524796

[B117] Wang S. Li L. Liu T. Jiang W. Hu X. (2020). miR-19a/19b-loaded exosomes in combination with mesenchymal stem cell transplantation in a preclinical model of myocardial infarction. *Regen. Med.* 15 1749–1759. 10.2217/rme-2019-0136 32772806

[B118] Xu B. Qin Y. Li D. Cai N. Wu J. Jiang L. (2020). Inhibition of PDE4 protects neurons against oxygen-glucose deprivation-induced endoplasmic reticulum stress through activation of the Nrf-2/HO-1 pathway. *Redox Biol.* 28:101342. 10.1016/j.redox.2019.101342 31639651 PMC6807264

[B119] Xu L. Liu T. Liu L. Yao X. Chen L. Fan D. (2020). Global variation in prevalence and incidence of amyotrophic lateral sclerosis: a systematic review and meta-analysis. *J. Neurol.* 267 944–953. 10.1007/s00415-019-09652-y 31797084

[B120] Xu Q. Zhao Y. Zhou X. Luan J. Cui Y. Han J. (2018). Comparison of the extraction and determination of serum exosome and miRNA in serum and the detection of miR-27a-3p in serum exosome of ALS patients. *Intractable Rare Dis. Res.* 7 13–18. 10.5582/irdr.2017.01091 29552440 PMC5849619

[B121] Yang D. Zhang W. Zhang H. Zhang F. Chen L. Ma L. (2020). Progress, opportunity, and perspective on exosome isolation – efforts for efficient exosome-based theranostics. *Theranostics* 10 3684–3707. 10.7150/thno.41580 32206116 PMC7069071

[B122] Yelick J. Men Y. Jin S. Seo S. Espejo-Porras F. Yang Y. (2020). Elevated exosomal secretion of miR-124-3p from spinal neurons positively associates with disease severity in ALS. *Exp. Neurol.* 333:113414. 10.1016/j.expneurol.2020.113414 32712030 PMC7502520

[B123] Zhang Y. Wang G. Wang T. Cao W. Zhang L. Chen X. (2019). Nrf2-Keap1 pathway-mediated effects of resveratrol on oxidative stress and apoptosis in hydrogen peroxide-treated rheumatoid arthritis fibroblast-like synoviocytes. *Ann. N. Y. Acad. Sci.* 1457 166–178. 10.1111/nyas.14196 31475364

[B124] Zhang Y. Yan T. Sun D. Xie C. Wang T. Liu X. (2020). Rutaecarpine inhibits KEAP1-NRF2 interaction to activate NRF2 and ameliorate dextran sulfate sodium-induced colitis. *Free Radic. Biol. Med.* 148 33–41. 10.1016/j.freeradbiomed.2019.12.012 31874248 PMC7376370

[B125] Zhao L. Hu C. Zhang P. Jiang H. Chen J. (2019). Preconditioning strategies for improving the survival rate and paracrine ability of mesenchymal stem cells in acute kidney injury. *J. Cell. Mol. Med.* 23 720–730. 10.1111/jcmm.14035 30484934 PMC6349184

[B126] Zheng Y. Wu G. Chen L. Zhang Y. Luo Y. Zheng Y. (2020). Neuro-regenerative imidazole-functionalized GelMA hydrogel loaded with hAMSC and SDF-1α promote stem cell differentiation and repair focal brain injury. *Bioact. Mater.* 6 627–637. 10.1016/j.bioactmat.2020.08.026 33005827 PMC7508914

[B127] Zhou Y. Zhao B. Zhang X. L. Lu Y. J. Lu S. T. Cheng J. (2021). Combined topical and systemic administration with human adipose-derived mesenchymal stem cells (hADSC) and hADSC-derived exosomes markedly promoted cutaneous wound healing and regeneration. *Stem Cell Res. Ther.* 12:257. 10.1186/s13287-021-02287-9 33933157 PMC8088044

[B128] Zhu D. Johnson T. K. Wang Y. Thomas M. Huynh K. Yang Q. (2020). Macrophage M2 polarization induced by exosomes from adipose-derived stem cells contributes to the exosomal proangiogenic effect on mouse ischemic hindlimb. *Stem Cell Res. Ther.* 11:162. 10.1186/s13287-020-01669-9 32321589 PMC7178595

[B129] Zhu S. Chen M. Deng L. Zhang J. Ni W. Wang X. (2020). The repair and autophagy mechanisms of hypoxia-regulated bFGF-modified primary embryonic neural stem cells in spinal cord injury. *Stem Cells Transl. Med.* 9 603–619. 10.1002/sctm.19-0282 32027101 PMC7180297

[B130] Zhu X. Zhang C. Shi M. Li H. Jiang X. Wang L. (2021). IL-6/STAT3-mediated autophagy participates in the development of age-related glomerulosclerosis. *J. Biochem. Mol. Toxicol.* 35:e22698. 10.1002/jbt.22698 33393185

[B131] Zuo X. Zhou J. Li Y. Wu K. Chen Z. Luo Z. (2021). TDP-43 aggregation induced by oxidative stress causes global mitochondrial imbalance in ALS. *Nat. Struct. Mol. Biol.* 28 132–142. 10.1038/s41594-020-00537-7 33398173

